# 661. In vitro Activity of Omadacycline and Comparator Antibiotics against Clostridioides difficile

**DOI:** 10.1093/ofid/ofac492.713

**Published:** 2022-12-15

**Authors:** Andrew M Skinner, Laurica A Petrella, Adam K Cheknis, Stuart Johnson

**Affiliations:** Loyola University Chicago Stritch School of Medicine, Maywood, Illinois; Edward Hines Jr VA Hospital, Hines, Illinois; Edward Hines Jr. VA Hospital, Hines, Illinois; Hines VA Hospital and Loyola University Medical Center, Hines, Illinois

## Abstract

**Background:**

Omadacycline was approved for the treatment of community acquired bacterial pneumonia (CABP) and acute bacterial skin and skin structure infections (ABSSSI) in 2018. Previous studies demonstrate that omadacycline has *in vitro* activity against *Clostridioides difficile.* We determined the *in vitro* activity of omadacycline and of comparator antimicrobials for the approved indications, CABP and ABSSSI, towards a contemporary and clinically relevant collection of *C. difficile* isolates.

**Methods:**

Antimicrobial agar dilution was performed on 200 clinical *C. difficile* isolates collected from 2014 – 2021. Isolates were selected based on the most prevalent restriction endonuclease analysis (REA) groups locally and nationally. Omadacycline was compared to 8 standard of care antimicrobials for CABP and ABSSSI: amoxicillin-clavulanate, azithromycin, ceftriaxone, clindamycin, doxycycline, linezolid, moxifloxacin, and trimethoprim-sulfamethoxazole.

**Results:**

Omadacycline demonstrated *in vitro* activity against all *C. difficile* isolates tested. The geometric mean MIC of omadacycline was 0.07 μg/ml and the MIC_50_ and MIC_90_ were 0.0625 μg/ml and 0.125 μg/ml, respectively. Among the comparator antibiotics, 52% of isolates were resistant to ceftriaxone and 37% of isolates were resistant to clindamycin. The majority of REA group BI isolates were resistant to clindamycin (77.7%, 28/36) and the clindamycin geometric mean MICs were higher for group BI compared to all other REA group strains (32 µg/ml and 5.33 µg/ml, respectively, p < 0.005). REA group BI also had elevated azithromycin and moxifloxacin geometric MICs (335.2 and 17.3 µg/ml, respectively). REA group DH isolates had higher trimethoprim-sulfamethoxazole geometric MICs compared to all other REA group strains (17.28 µg/ml and 8.14 µg/ml, respectively p >0.001). Nearly half of REA group BK isolates (47%) had MICs ≥2 µg/ml which did not correspond to elevated omadacycline MICs for the same isolates.

**Conclusion:**

Omadacycline demonstrated consistently low MICs against *C. difficile* when compared to approved antimicrobials for CABP and ABSSI which varied in activity among particular *C. difficile* strains. Omadacycline may reduce the risk of developing a *C. difficile* infection and requires further study.

**Disclosures:**

**Stuart Johnson, M.D.**, Ferring Pharmaceuticals: Membership on Ferring Publication Steering Committee|Ferring Pharmaceuticals: Employee|Summit Plc: Advisor/Consultant.

